# Comparison of success rate and onset time of two different anesthesia techniques

**DOI:** 10.4317/medoral.20526

**Published:** 2015-04-10

**Authors:** Abbas Haghighat, Zahra Jafari, Dariush Hasheminia, Mohammad-Hasan Samandari, Vajihe Safarian, Amin Davoudi

**Affiliations:** 1 First author-Assistant professor of Oral and Maxillofacial Surgery, Dental Implants Research Center, Department of Oral and Maxillofacial Surgery, School of Dentistry, Isfahan University of Medical Sciences, Isfahan, Iran; 2 Corresponding author- Dentist, Torabinejad Dental Research Center, Isfahan University of Medical Sciences, Isfahan, Iran; 3 Assistant professor of Oral and Maxillofacial Surgery, Torabinejad Dental Research Center, Department of Oral and Maxillofacial Surgery, School of Dentistry, Isfahan University of Medical Sciences, Isfahan, Iran; 4 Assistant professor of Oral and Maxillofacial Surgery, Dental Materials Research Center, Department of Oral and Maxillofacial Surgery, School of Dentistry, Isfahan University of Medical Sciences, Isfahan, Iran; 5 Dentist, Torabinejad Dental Research Center, Isfahan University of Medical Sciences, Isfahan, Iran; 6 Dentistry student, Dental Students Research Center, School of Dentistry, Isfahan University of Medical Sciences, Isfahan, Iran

## Abstract

**Background:**

Using local anesthetic is common to control the pain through blocking the nerve reversibly in dental procedures. Gow-Gates (GG) technique has a high success rate but less common. This study aimed to compare the onset time and success rate in GG and standard technique of inferior alveolar nerve block (IANB).

**Material and Methods:**

This descriptive, single blind study was consisted of 136 patients (59 males and 77 females) who were randomly received GG or IANB for extraction of mandibular molar teeth. Comparisons between the successes of two anesthetic injection techniques were analyzed with Chi-square test. Incidence of pulpal anesthesia and soft tissue anesthesia were analyzed with Kaplan-Meier method. Mean onset times of pulpal anesthesia, soft tissue and lip numbness were analyzed with Log-Rank test. Comparisons were considered significant at *P*≤0.05 by using SPSS software ver.15.

**Results:**

The incidence of pulpal anesthesia in the IANB group (canine 49.3%, premolar 60.3%) were not significantly different from the GG group (canine 41.3%, premolar 74.6%) (*P*=0.200 and *P*=0.723). The success rate in the IANB group (80.82%) was not significantly different from the GG group (92.02%) (*P*=0.123). Furthermore, onset time of lip and buccal soft tissue numbness in GG group (3.25, 4.96 minutes) was quite similar to IANB group (3.22, 4.89 minutes) (all *P*values >0.05).

**Conclusions:**

Although this study demonstrated higher clinical success rate for GG than IANB technique, no significant differences in success rates and onset time were observed between two techniques.

**Key words:** Anesthesia, Inferior alveolar nerve, nerve block, success rate.

## Introduction

The use of local anesthetic solutions is very common for controlling pain in dental procedures. They reduce the pain through blocking the nerve reversibly. In dental procedures, cartridge is used for local anesthesia which contains: lidocaine 2% (as a super effective medicine which is sustainable to high quality autoclaves and boiling (1) and vascular constriction such as adrenalin to reduce toxicity of drug absorption and long-term effects of local anesthetic (2,3).

Deep anesthesia is very important particularly in dental surgery. Operator (choice of anesthetic technique) and patient (anatomical, pathological or psychological) are known as the main factors in anesthetic success or failure rates. According to Lopez *et al*. failure is defined as “If symptoms of anesthesia are not identified after a prudent period of 10-15 minutes following the anesthetic procedure” (4).

Maxillary anesthesia is mostly successful except for the cases with abnormal anatomies or pathologic conditions. Maxillary teeth apexes are not surrounded by dens bone and expert operators mostly can anesthetize the nerves’ root in high success rate, easily. But this condition in adults’ mandibular is different in which success rate in pulpal anesthesia is low and much more difficult because higher density of cortical alveolar bone in mandibular teeth (5).

There are some factors which affect the inferior alveolar nerve blocks success rate including: patient fear of receiving the anesthetic drug, systemic and local complication of intraoral injection, biologic diversity responsible to the drugs, anatomical variations, infections and inflammations, intra-vein injections and needle deflection, dens bone, bifid mandibular nerve, accessory mental foramen, anastomoses, expired solution and incorrect method of injection (4-8).

In addition, there are three injection techniques for local anesthesia in mandibular restorative and surgical procedures: The inferior alveolar nerve block (IANB), Gow-Gates (GG) and Vazirani-Akinosis techniques.

IANB is the common injection technique for attaining local anesthesia in mandibular jaw. However the IANB does not always result in successful pulpal anesthesia (9). Failure rates of 10-39% have been reported (5).

In 1973 Gow-Gates used extraoral landmark for mandibular anesthesia. In this new technique the target site is the neck of the mandibular condyle (10). Hung *et al*. (11) and Goldberg *et al*. (12) used pulp tester to evaluate anesthesia and found no difference between the IANB and GG techniques, while Agren and Danielsson (13) found faster time of pulpal anesthesia for GG technique.

Some studies have shown higher success rates with the GG technique (95-96%) versus the conventional IANB (65-79%) during surgery (5,14,15), but Todorvice *et al*. (16) found the conventional IANB was better than the GG technique.

Some studies found that the success rate of lip numbness in conventional IANB was similar to the GG technique (11,12,15) 

This study attempted to compare the onset time, success rate and degree of pulpal anesthesia obtained from a conventional IANB and GG by using 2% lidocaine with 1:80,000 epinephrine in vital teeth.

## Material and Methods

- Ethics: The study was approved by the local ethics committee and conducted in accordance with the rules of Isfahan University of Medical Sciences. An informed consent was signed by patients, and the study has been ethically approved by Isfahan University of Medical Sciences, dental school.

During this prospective randomized clinical study, 136 patients were studied. The patients admitted to the Orofacial Surgery Department of the University of Medical Sciences, Isfahan, Iran.

Sample size calculations required up to120 adults which 136 adults participated (59 men and 77 women) in this study and each selected case required extraction of mandibular molars. The patients were between the ages of 15 and 50 years old without taking any drugs that would alter their perception of pain (opioids and non-estroidal anti-inflammation). In addition, they did not show any inflammations in injection site. Exclusion criteria were: periodontal diseases, physical and mental retardation, being younger than 15 and older than 50years of age, inability of enough mouth opening for GG injection, deep caries and large restorations in mandibular canine and premolar in side of injection.

The total of 136 patients divided in two groups: 63 patients for GG group and 73 patients for IANB group.

The study operators were specialists and residents of Orofacial Surgery Department of dentistry faculty who standardized their injection skills for two mandibular block methods.

For the injection purposes this study used standard syringes equipped with a blood aspiration device (Novocol, USA), fitted with a 27-gauge 0.4 × 35mm needle (Denject, Korea), 2% lidocaine with 1:80,000 epinephrine (Daroopakhsh, Iran).

- Before injection 

The chosen teeth for experiment were first premolar and canine. The contra lateral canine was as the anesthetized control to certify the accuracy of the pulp tester operating. Clinical experiments revealed that all the teeth were free of large restoration, caries, and periodontal disease, also participants’ records showed no trauma or sensitivity.

Cotton rolls were used to isolate contra lateral canine and experimental teeth, while toothpaste was applied to probe tip (which was then placed midway between the gingival margin and the occlusal or incisal edge of the tooth) after that the teeth were tested by using Electrical Pulp Tester (Parkell, USA) to record baseline vitality. The current rate was increased from minimum (0) to the maximum (8). The number associated with the initial sensation was recorded.

Furthermore, the neurosensory testing was performed on the patients’ lips to ensure the accuracy of light touch sensitivity before any injections (a soft hair brush drawn gently on the patients’ lower lip).

After finishing the test stage, The Orofacial specialists injected Lidocain 2% by using GG or IANB techniques to block mandible within 60-90 seconds. The operators aspirated during injection and positive or negative condition was recorded. After injection the timer was turned on.

For IANB technique, 1.5 ml anesthetic solution deposited during a period of 60-90 second and when the needle was withdrawn remaining anesthetic solution injected for lingual nerve anesthesia. In addition, 0.5 ml anesthetic solution injected for long buccal anesthesia.

For GG technique, 1.8ml anesthetic solution was deposited during a period of 60-90 seconds. After the injection, the subject was asked to keep his/her mouth wide open for 1 minute.

The onset of lower lip anesthesia tested with using a soft hair brush by drawing it gently on the patients’ lower lip, the patients also were asked about lip numbness. Within first five minutes after injection, the lips’ sensation in the lateral side of the lower lip was assessed and if this sensation was not present in this period, another five minutes was allocated to lapse and checked every minutes. Also failure of lower lip anesthesia was reported, if lips’ sensation was not altered after 10 minutes (12).

The buccal soft tissue was tested with tongue blade every two minutes for 10 minutes, and onset of soft tissue anesthesia was recorded in this period. At the same time, pulpal anesthesia of canine and premolar were tested by electrical pulp tester every three minutes after injection for 10 minutes. The negative responsible to number 8: EPT was assumed as successful pulpal anesthesia (13).

The patients were requested to report any pain during the surgery or extraction procedure (soft tissue cutting, bone cutting, tooth extraction). A verbal analogue scale was engaged to evaluate the pain intensify during surgery ranging from 0: no pain; 1: mild and bearable pain; 2: moderate pain; 3: unbearable and severe pain.

According To this classification, 0 and 1 were assumed as injection success, whereas 2 and 3 manifested the failure of injection (17).

Two injection techniques for incidence of lip numbness, soft tissue anesthesia incidence, incidence of pulpal anesthesia, and anesthetic success were analyzed and compared by using SPSS ver.15. The mean onset time of pulpal anesthesia, lip numbness and soft tissue anesthesia were analyzed by Log rank. Comparisons were considered significant at *P*≤0.05.

## Results

The total of 136 adult subjects, 59 men and 77 women, aged 15-50 years old (average 25 years old) who needed extraction of mandibular molars participated. The onset time and incidence of lip numbness and soft tissue anesthesia ( Table 1) revealed that there were no significant difference in lip numbness (*P*=0.268) and soft tissue anesthesia (*P*= 0.878) between the IANB and GG techniques.

**Table 1:**
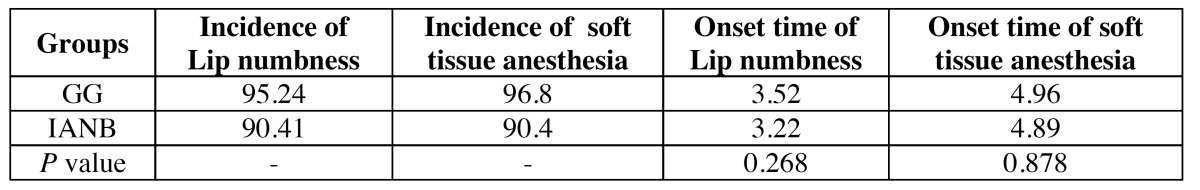
Incidence and mean onset time of soft tissue anesthesia and lip numbness.

This study used the electrical pulp tester for examining the pulpal anesthesia. Based on the analyses, IANB technique (7.7-8.8 min) anesthetized the pulp faster than the GG technique (8.3-9.3 min), but no significant difference was observed (*p*>0.05).

The incidence of pulpal anesthesia represented no significant difference between the IANB and GG techniques in first 10 minutes ( Table 2).

**Table 2:**
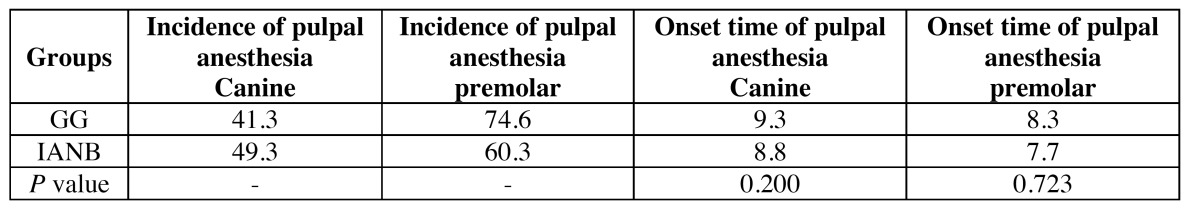
Incidence and mean onset time of pulpal Anesthesia.

In this study, the success rate of GG technique was 92%, while the percentage was recorded 80.8% for IANB technique. Severity of pain showed no significant difference between IANB and GG techniques (*P*=0.123) ( Table 3).

**Table 3:**
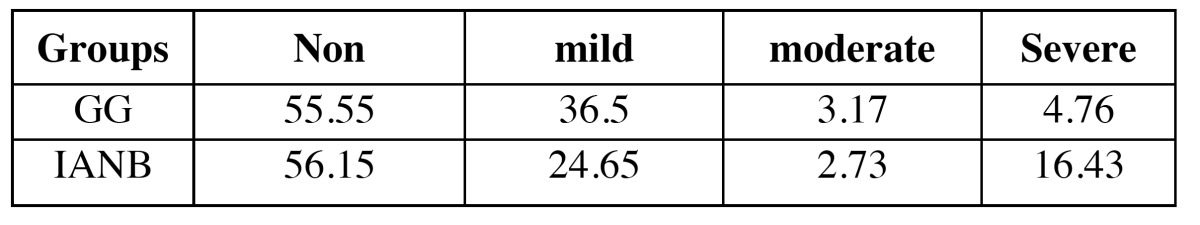
Pain rate and Success rate of Anesthesia technique between groups.

## Discussion

This study showed no significant differences between the IANB and GG techniques in terms of onset time and success rate.

The results revealed that the success rate of lip numbness in GG technique was higher than IANB technique, but there were no significant difference between two methods. Sisk AL reported a higher success of GG technique too (15).

It is believed that anesthesia success rate is higher in GG because of higher constancy of landmarks used to guide placement and insertion of the local anesthetic needle in comparison with IANB. In addition, the diverse anatomy of patients in the locations of the mandibular foramen and lingual decrease the success rate IANB technique.

Hung *et al*. (11) and Goldberg *et al*. (12) in two separate studies demonstrated that all subjects achieved 100% lip numbness with both GG and IANB techniques, but this percentages were 95% and 92% for GG and IANB respectively in present study. It should be noted that this study examined the patient only for 10 minutes, while Goldberg *et al*. (12) examined the patient for 21 minutes.

Furthermore, onset time of lip numbness in GG technique was slower than the IANB in this study, but no significant difference between two methods recorded. Sisk also revealed the onset time of lip numbness in GG group was slower because in this area the size of nerve fibers are larger and distance from the injection site is far (15).

In the current study, onset time for IANB of lip numbness was 3.2 minutes. Also, Waikakul and Punwutikorn reported 3 minutes onset time of lip numbness for the same method (18).

In this study success rate of soft tissue anesthesia manifested no differences between GG and IANB techniques. Goldberg *et al*. (12) and Hung *et al*. (11) reported no difference between two methods too. 1.8 ml lidocaine was derived in GG and IANB groups (0.5 ml was administered for long buccal technique). Gow-Gates believed that for the GG technique there is no need for buccal infiltration. Goldberg *et al*. used 3.6 ml lidocaeine for both techniques (12), while Hung *et al*. used 2.7 ml for both groups (11). It seems that the increment of anesthetic solution does not affect soft tissue anesthesia.

Some studies used the electrical pulp tester (11,12,19) as present study. According to the results, there was no significant difference between the IANB and GG techniques, our study supports the finding of the study by Goldberg *et al*. (12), Hung *et al*. (11) too.

In general, the success rate of pulpal anesthesia for first premolar in IANB method (60%) is similar to findings of Nusstein *et al*. (61%) (9) and Goldberg *et al*. (62%) (12).

Some study reported faster onset time of pulpal anesthesia for IANB than GG techniques (12,13). However, in this study onset time of pulpal anesthesia in GG was faster but difference was not statistically significant.

In this study, patient were examined for 10 minute after the injection, therefore faster onset time of IANB does not affect the overall success rate of IANB technique.

In some studies such as Agren and Danielsson (13) the reason of slower onset time of GG technique was explained due to the injection site which was far from the inferior alveolar nerve. Also, previous studies reported slower onset of anesthesia for GG technique (13,16,20).

Although the success rate of GG (92%) was higher than the IANB technique (80.8%) in present study, the difference was not statistically significant. Some studies have concluded that the success rate of GG (95%-96%) is higher than the IANB technique (65%-79%) during surgery (13-15). Todorvice *et al*. reported the success rate of IANB is higher than the GG technique during the extraction (16).

Robertson (21) and Gow-Gates (10) examined two methods during restorative procedure and they found that the success rate of GG (92%-98%) was higher than the IANB technique (71%-86%).

## Conclusion

The individuals were young adults with a mean age of 25 years in present study, so the results cannot be generalized to older or younger people.

The results of this study were not comparable to other previous studies as the extraction and variations in restorative procedure might not be considered as a good criteria for pulpal anesthesia.

Although this study demonstrated higher clinical success rate of GG than IANB technique, success rates and onset time are not significantly different in IANB and GG techniques.
